# Supplementary material for: Empirical methods for controlling false positives and estimating confidence in ChIP-Seq peaks
Source: BMC Bioinformatics. 2008 Dec 5;9:523. doi: 10.1186/1471-2105-9-523 (PMC2628906; doi:10.1186/1471-2105-9-523)
Supplement: Additional File 1 — A variety of html documents from the USeq web site detailing the available applications, their best usage, output file type descriptions, command line menus, etc. [file 1471-2105-9-523-S1.zip › USeqUserGuides/JavaDoc.zip!/JavaDoc/gata/aligner/class-use/AlignParams.html]

Uses of Class gata.aligner.AlignParams


|  |  |  |
| --- | --- | --- |
| **Overview** \| **Package** \| **Class** \| **Use** \| **Tree** \| **Deprecated** \| **Index** \| **Help** | |  |
| PREV   NEXT | **FRAMES**    **NO FRAMES**     **All Classes** |  |


---


## **Uses of Class gata.aligner.AlignParams**

| Packages that use AlignParams | |
| --- | --- |
| **gata.aligner** | For aligning and post processing DNA sequences using BLAST for subsequent import into GATAPlotter. |
| **gata.main** | Main app, utility, and params for GATAPlotter. |
| **gata.plotter** | GATAPlotter primary panel, sliders, tools, and conservation panels. |

Uses of AlignParams in gata.aligner

| Fields in gata.aligner declared as AlignParams | |
| --- | --- |
| `AlignParams` | `Blast.ap` |

| Methods in gata.aligner that return AlignParams | |
| --- | --- |
| `AlignParams` | `LocalAlignment.getAP()` |

Constructors in gata.aligner with parameters of type AlignParams

`Alignment(java.lang.String s1,
int start1,
int stop1,
java.lang.String s2,
int start2,
int stop2,
int aScore,
int ori,
AlignParams ap,
LocalAlignment la)`

`Blast(AlignParams AP)`

`LocalAlignment(java.lang.String seq_1,
int startSeq_1,
int stopSeq_1,
java.lang.String seq_2,
int startSeq_2,
int stopSeq_2,
int ori_,
AlignParams ap_,
int score_)`

`Seqs(AlignParams ap,
MultiFastaParser ref,
MultiFastaParser comp,
int index)`

Uses of AlignParams in gata.main

| Methods in gata.main that return AlignParams | |
| --- | --- |
| `AlignParams` | `GATAParams.getAlignParams()` |

| Methods in gata.main with parameters of type AlignParams | |
| --- | --- |
| `void` | `GATAParams.setAlignParams(AlignParams ap)` |

Uses of AlignParams in gata.plotter

| Methods in gata.plotter with parameters of type AlignParams | |
| --- | --- |
| `static boolean` | `MultiAlignPanel.checkAlignParams(AlignParams[] alignParams)`             Checks to see if the same params were used used in making the alignments, note score cut off is not checked |
| `static int` | `MultiAlignPanel.fetchMinScore(AlignParams[] alignParams)`             Finds lowest minScore in an array of AlignParams. |

Constructors in gata.plotter with parameters of type AlignParams

`Sliders(AlignParams ap,
GATAParams params)`

---


|  |  |  |
| --- | --- | --- |
| **Overview** \| **Package** \| **Class** \| **Use** \| **Tree** \| **Deprecated** \| **Index** \| **Help** | |  |
| PREV   NEXT | **FRAMES**    **NO FRAMES**     **All Classes** |  |


---
